# Pathogenic Roles of Macrophage Migration Inhibitory Factor during Dengue Virus Infection

**DOI:** 10.1155/2015/547094

**Published:** 2015-03-02

**Authors:** Yung-Chun Chuang, Hong-Ru Chen, Trai-Ming Yeh

**Affiliations:** ^1^Department of Medical Laboratory Science and Biotechnology, Medical College, National Cheng Kung University, Tainan 70101, Taiwan; ^2^Center of Infectious Disease and Signaling Research, Medical College, National Cheng Kung University, Tainan 70101, Taiwan; ^3^The Institute of Basic Medical Sciences, Medical College, National Cheng Kung University, Tainan 70101, Taiwan

## Abstract

Dengue virus (DENV) infection is the most common cause of viral hemorrhagic fever, which can lead to life-threatening dengue hemorrhagic fever/dengue shock syndrome (DHF/DSS). Hemorrhage and plasma leakage are two major hallmarks of DHF/DSS. Because the mechanisms causing these pathogenic changes are unclear, there is no effective therapy against DHF/DSS. In this review, we focus on the possible pathogenic effects of a pleiotropic cytokine, macrophage migration inhibitory factor (MIF), on the pathogenesis of DENV infection. MIF is a critical mediator of the host immune response and inflammation, and there is a correlation between the serum levels of MIF and disease severity in dengue patients. Furthermore, MIF knock-out mice exhibit less severe clinical disease and lethality. However, the role of MIF in the pathogenesis of DHF/DSS is not limited to immune cell recruitment. Recent evidence indicates that DENV infection induced MIF production and may contribute to vascular hyperpermeability and viral replication during DENV infection. The expression of both adhesion and coagulation molecules on MIF-stimulated monocytes and endothelial cells is also increased, which may contribute to inflammatory and anticoagulatory states during DHF/DSS. Therefore, blocking MIF production or its function may provide a solution for the treatment and prevention of DHF/DSS.

## 1. Introduction

### 1.1. The Structure and Expression of Macrophage Migration Inhibitory Factor (MIF)

Macrophage migration inhibitory factor (MIF), which is also known as glycosylation-inhibiting factor (GIF), L-dopachrome isomerase, or phenylpyruvate tautomerase, was first identified as a cytokine inhibiting the random migration of macrophages [1, 2]. MIF is an evolutionarily highly conserved protein that is abundantly expressed in human and other species. MIF is composed of 114 amino acids, producing a cytokine of 12.5 kDa [3]. In contrast to other cytokines, MIF possesses a unique catalytic function as a tautomerase. Under physiologic conditions, MIF exists as a trimer consisting of three identical subunits, an arrangement that confers a three-dimensional structure of MIF resulting in a catalytic site located in the intermonomeric pocket [4]. Although T cells were first identified as the main source of this cytokine, MIF is now known to be widely expressed in various cell types, including monocytes, macrophages, hepatocytes, and endothelial cells [1, 2, 5–8]. The secretion of MIF by macrophages is induced by low levels of glucocorticoids and is suggested to counteract the inhibitory effects of glucocorticoids in the regulation of the immune system [9–11]. Recently, it has been revealed that activated platelets are also a source of MIF [12].

### 1.2. The Activating Mechanism of MIF

Despite its wide tissue distribution, the secretion of MIF is tightly regulated by relevant triggers, such as inflammation and hypoxia. It has long been known that the secretion of MIF is correlated to infectious diseases, autoimmune diseases, heart and vascular diseases, and cancer. After secretion, MIF activates downstream pathways in an autocrine or paracrine manner. The first identified receptor of MIF was CD74, the membrane-expressed form of invariant chain and an MHC class II chaperone [13]. Due to the lack of an intracellular domain, the activation of CD74 by MIF relies on the recruitment of coreceptors such as CD44 or CXCR2 and CXCR4 [14]. In a recent study, another chemokine receptor, CXCR7, has been shown to engage with MIF to modulate tumor metastasis [15]. CD44 is required for transmitting the MIF/CD74 signal by relaying the Src tyrosine kinase-mediated phosphorylation of serine on the cytosolic tail of CD74 and CD44; this phosphorylation then activates the downstream ERK/MAPK and PI3K/Akt pathways [16–18]. In addition to CD74, the direct binding of MIF and CXCR2 or CXCR4 was also observed to induce calcium influx and the rapid activation of integrins by Gi-coupling [19]. CXCR7 could be activated by MIF to initiate the Akt pathway to regulate platelet apoptosis [20]. In addition to transmitting signals through receptors, MIF can be endocytosed into the cytosol and interact with JAB-1 to inhibit the activity of AP-1 proteins [21]. Secreted MIF is capable of activating T cells and macrophages to produce proinflammatory cytokines, including tumor necrosis factor- (TNF-) *α*, interleukin- (IL-) 1*β*, IL-2, IL-6, IL-8, and interferon- (IFN-) *γ*, leading to inflammatory responses. Therefore, MIF plays a pivotal role in the modulation of both innate and adaptive immune responses [8, 22].

### 1.3. The Role of MIF in Inflammatory Diseases

The first evidence implicating the pathogenic roles of MIF in inflammatory diseases dates back to two decades [23]. Employing a mouse endotoxic shock model, recombinant MIF was found to greatly enhance lethality when coinjected with lipopolysaccharide (endotoxin), whereas an anti-MIF antibody conferred full protection against lethal endotoxemia. Elevated levels of MIF were subsequently found in the plasma of patients with sepsis and septic shock and were positively correlated with the sepsis severity [24, 25]. Blockage of MIF with neutralizing antibodies or the tautomerase inhibitor 4,5-dihydro-3-(4-hydroxyphenyl)-5-isoxazole acetic acid, methyl ester (ISO-1) increases the survival rate in septic mice models [26, 27]. In addition to sepsis, MIF knock-out (*Mif* ^−/−^) mice show reduced mortality compared to wild-type mice after* Toxoplasma gondii* infection, indicating that MIF is also involved in the pathogenesis of infection by this protozoan [28]. In addition to the pathogenic roles of MIF in acute infection, MIF is also essential for the pathogenesis of chronic diseases, such as autoimmune and cardiovascular diseases, as well as cancer [29–33]. However, unlike the case in cancer and autoimmune diseases, MIF may have a protective effect in the heart during ischemia or other cardiovascular diseases [31, 34].

### 1.4. MIF in Viral Infection

In addition to bacterial infection, elevated MIF levels are also observed in viral infections, such as those caused by influenza virus, human immunodeficiency virus (HIV), Ebola virus, and dengue virus (DENV) [35–39]. DENV infection generally causes mild symptoms such as fever, headache, and muscle and joint pain, comprising dengue fever (DF). In some cases, especially during secondary infection with a different serotype of DENV, DENV infection may progress to dengue hemorrhagic fever (DHF) or dengue shock syndrome (DSS) [40]. DHF is a severe febrile disease characterized by abnormalities in homeostasis and increased capillary leakage that can progress to hypovolemic shock (DSS). Although the mechanisms causing hemorrhage and vascular leakage in DHF/DSS remain unclear, there are few hypotheses for severe dengue disease, the most well known of which is antibody-dependent enhancement (ADE) [41]. According to ADE, the antibodies generated in the first DENV infection have no protective effect but rather enhance the second infection of a different DENV serotype through the Fc*γ* receptor of such host cells as macrophages. Other hypotheses, including cytokine production and complement activation, and autoantibody immunopathogenesis also play crucial roles that might contribute to severe DHF/DSS during DENV infections [42–44]. Nevertheless, it is known that the serum levels of MIF in dengue patients correlate with disease severity and clinical outcome [39]. Furthermore,* Mif* ^−/−^ mice exhibit less severe clinical disease, lower viremia, and lower viral load in the spleen than wild-type mice after DENV infections [38]. Blocking MIF also rescues DENV-induced vascular leakage, indicating the importance of MIF in the pathogenesis of DENV infection [45]. Therefore, the current review focuses on the possible pathogenic roles of MIF during DENV infection.

## 2. MIF Production Induced by DENV Infection

DENV infection, but not stimulation with UV-inactivated DENV, induces MIF secretion in a dose- and time-dependent manner in the human epithelial cell line A549 [46]. A MIF promoter assay and reverse transcription polymerase chain reaction (RT-PCR) demonstrate that MIF gene transcription is activated during DENV infection. Furthermore, DENV infection induces NF-*κ*B activation, and the DENV-induced production of MIF is inhibited in the presence of the NF-*κ*B inhibitor dexamethasone or curcumin. In addition, different cell types, such as primary human vascular endothelial cells (HUVECs) and peripheral blood mononuclear cells (PBMCs), have different abilities to release MIF after DENV infection. Interestingly, DENV infection of the human monocytic cell line THP-1 and PBMCs and the subsequent MIF production are enhanced in the presence of antibodies against DENV. This phenomenon may due to an ADE effect. Based on ADE, antibodies against DENV may enhance virus uptake by macrophages through the Fc receptor, thus enhancing viral replication and altering the cytokine production profile [47]. Therefore, it is possible that DENV infection of human cells induces NF-*κ*B activation, which leads to MIF production and that this process is enhanced in the presence of preexisting anti-DENV antibodies.

## 3. MIF in DENV Replication

It is known that MIF is involved in the replication of many different viruses during infection. For example, in HIV-1 infection, MIF neutralization can diminish HIV-1 replication in human PBMCs [48] (Table 1). In* Mif* ^−/−^ mice, West Nile virus (*WNV*) and Ross River virus (RRV) have shown reduced viral replication in the brain, ankle, serum, and spleen [49, 50]. However, there is only one related reference to date indicating the importance of MIF in DENV replication [38]. In addition to lower levels of TNF-*α*, IL-6, and PEG_2_, the viral load of DENV in* Mif* ^−/−^ mice is lower than that in wild-type mice. Moreover, the mortality of DENV-infected mice is delayed and the thrombocytopenia is reduced in* Mif* ^−/−^ mice. However, the precise mechanism by which MIF regulates DENV replication is still unknown.

Several studies have indicated that autophagy is required for optimal DENV replication [51–53] and different cytokines have different effects on autophagy formation. T helper type 1 (Th1) cytokines such as IFN-*γ*, IL-12, and TNF-*α* induce or promote autophagy in macrophages as well as nonimmune cells [54, 55]. In contrast, Th2 cytokines such as IL-4, IL-10, and IL-13 appear to be antagonists of autophagy induction [56]. Because MIF can induce Th1 cytokine expression, it may contribute to autophagy formation. Recently, we found that MIF could enhance autophagy via reactive oxygen species (ROS) generation [57]. Thus, autophagy may be one of the possible pathways by which MIF may regulate DENV replication. However, whether MIF-induced autophagy plays a pivotal role in DENV replication remains to be proven.

## 4. MIF in DENV-Induced Vascular Hyperpermeability

Although elevated MIF levels were observed in diseases that feature vascular leakage, the regulation of vascular permeability by MIF was first indicated in a study of WNV infection [50]. This study suggested that brain infection with WNV might be promoted by MIF-induced increased permeability of the blood-brain barrier. MIF-induced vascular leakage was later suggested in DENV infection [45]. Recombinant MIF as well as a DENV infection-induced conditioned medium increases vascular permeability by inducing disarray of the tight junction protein ZO-1, effects that are blocked by a MIF inhibitor (ISO-1) or CD74 knockdown. However, the mechanism by which MIF regulates the alignment of junction proteins remains unclear. Cytokines increase vascular permeability by regulating the expression and localization of junction proteins by various mechanisms. For example, VEGF induces endothelial hyperpermeability through the endocytosis of junction proteins, TNF-*α* alters vascular permeability by decreasing the mRNA content related to junction proteins, and IL-1*β* and thrombin induce the translocation of junction proteins and increase vascular permeability by regulating the arrangement of the cytoskeleton [58–62]. MIF was previously demonstrated to induce autophagy in hepatocytes [57]. Although autophagy has been suggested to regulate the turnover of junction proteins, whether it mediates vascular permeability has not yet been studied. Our recent work suggested that MIF might regulate vascular permeability through autophagy [63]. Further investigation of how MIF and autophagy regulate vascular permeability will open up promising novel therapeutic avenues to prevent DENV-induced vascular leakage.

## 5. MIF in DENV-Induced Adhesion Molecule and Coagulation Molecule Expression

It is known that MIF can induce the production of intercellular adhesion molecule-1 (ICAM-1) [64] and the coagulation molecule thrombomodulin (TM) [65]. ICAM-1 is a ligand for lymphocyte function-associated molecule-1 (LAF-1), which is expressed on the leukocyte cell surface. Through ICAM-1 and LFA-1 interaction, leukocytes can bind to endothelial cells and then transmigrate into tissues. In addition, the production of coagulation and anticoagulation factors is affected by MIF in endothelial cells, THP-1 cells and Hep G2 cells [66]. Reverse transcription-polymerase chain reaction (RT-PCR), immunofluorescent staining, and flow cytometry demonstrate that recombinant MIF induces TM protein expression in endothelial cells and THP-1 cells, and this effect is blocked by a MIF-neutralizing antibody [66]. TM is a transmembrane glycoprotein that is expressed on the surface of vascular endothelial cells as well as monocytes and a variety of other cell types. TM can compete with fibrinogen to bind to thrombin and inhibit fibrin formation, thereby interfering with coagulation. The thrombin-TM complex can also activate protein C (APC), which can digest the active clotting factors Va and VIIIa to inhibit further thrombin formation. Thus, TM plays an important role in the anticoagulation pathway. Interestingly, the serum level of TM is also correlated with disease severity in dengue patients, suggesting that the expression of TM may contribute to hemorrhage in DHF/DSS patients [67]. Taken together, the results of studies suggest that DENV infection induces MIF production, which in turn stimulates monocytes or endothelial cells to express TM and ICAM-1. Thus, MIF may play important roles not only in recruiting inflammatory cells but also in regulating coagulation.

## 6. Conclusion

**Figure 1:**
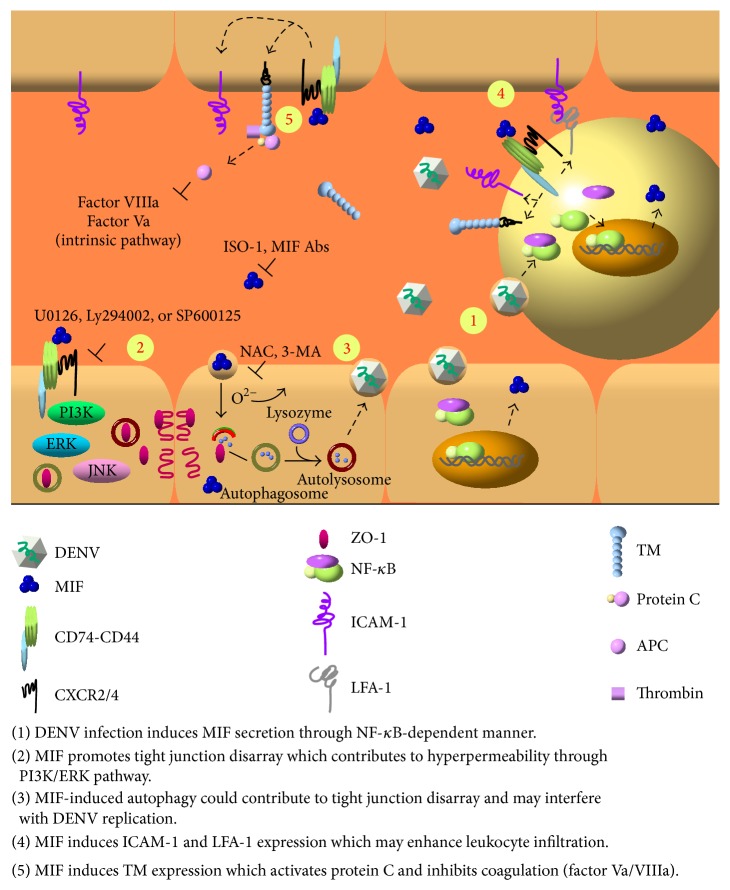
Schematic diagram of the pathogenic roles of MIF and possible strategies to block its effects during DENV infection. MIF expression is upregulated during DENV infection. Blocking MIF function by a MIF inhibitor (ISO-1) or MIF antibodies or its signal transduction by U0126 (Erk inhibitor), Ly294002 (PI3K inhibitor), or SP600125 (JNK inhibitor) may prevent MIF-induced permeability. In addition, MIF-induced autophagy may enhance DENV replication; therefore, blocking autophagy by NAC (ROS scavenger), 3-MA (autophagy inhibitor), or a MIF inhibitor (ISO-1) may reduce DENV replication. In addition, MIF induces the upregulation of ICAM-1 and LFA-1 expression, which may influence leukocyte recruitment. Finally, TM expression induced by MIF may activate protein C and inhibit thrombin formation. These effects of MIF can be blocked by its inhibitor or an antibody.

DENV infection poses a great threat to human health, with a considerable economic impact on tropical and subtropical areas of the world. Currently, there is no vaccine or specific drug to prevent or treat the disease. Indeed, the only means for preventing a DENV pandemic rely largely on targeting the vectors, which has limited effectiveness. The findings mentioned above suggest that DENV infection induces MIF production and secretion, which is NF-*κ*B-dependent, and secreted MIF can enhance DENV replication and increase vascular leakage through autophagy. In addition, MIF can regulate adhesion molecule and coagulation molecule expression on the surface of endothelial and immune cells, which may contribute to inflammation and hemostatic abnormality during DENV infection. Therefore, prevention of the production of MIF or blocking its function by small molecules or anti-MIF antibodies may represent an additional approach to prevent the development of DHF/DSS (Figure 1).

The therapeutic antagonism of cytokines such as TNF-*α* or IL-1 by biological agents has been indicated to be a successful strategy in certain autoimmune diseases. Because MIF is involved in a diversity of inflammatory diseases, it has the potential to be a therapeutic target in different inflammatory diseases and is not disease-specific. Moreover, compounds binding to the catalytic site of MIF that could be active anti-MIF drug candidates have been reported [68]. If the potential of such compounds is proven in clinical study, these compounds could be manufactured at relatively low cost compared to biological agents, which may benefit not only patients with DENV infection but also those with other inflammatory diseases.

## Figures and Tables

**Table 1:** Effects of MIF in the replication of different viruses.

Virus	Model	Treatment	Viral replication	Reference
HIV-1	HIV-1-infected PBMCs	Anti-MIF antibodies rMIF	p24 expression ↓ p24 expression ↑	[48]

WNV	WNV-infected *Mif* ^−/−^ mice		WNV E-mRNA ↓ in brain tissues	[50]

RRV	RRV-infected *Mif* ^−/−^ mice		Virus PFU ↓ in ankle tissues	[49]

DENV	DENV-2-infected *Mif* ^−/−^ mice		Virus PFU ↓ in serum and spleen	[38]
